# Association between pelvic inflammatory disease and abortions

**DOI:** 10.4103/2589-0557.75030

**Published:** 2010

**Authors:** Sangita V. Patel, Rajendra K. Baxi, Prakash V. Kotecha, Vihang S. Mazumdar, Kedar G. Mehta, Mansi Diwanji

**Affiliations:** Department of Preventive and Social Medicine, Medical College, Baroda, Gujarat, India

Sir,

Pelvic inflammatory disease (PID) is one of the most serious infections facing women today. It is a common problem encountered in gynecologic infertility, family planning, postnatal, legal abortions, and sterilization clinics in India and abroad.[1]

The identification of the risk factors associated with PID is crucial to efforts for the prevention of these consequences.[2]

Wasserheit estimated that in Southeast Asia, postabortion infection is the leading cause of PID, and in Pakistan, 23% of PID relate to unsafe abortion.[3] So the case–control study was conducted to know the association between PID and abortion.

A total of 150 patients of PID who attended the gynecology OPD of Shree Sayaji General Hospital (SSGH) over a period of 1 year were selected with uniformly accepted criteria for PID as given under: complain of lower abdominal pain, vaginal discharge, and adenexal tenderness leading to pain. All cases meeting with the above diagnostic criteria were labeled as clinical cases of PID by the gynecologist. After taking consent, we obtained information by conducting in-depth interviews up to three sessions with each patient. For each case, a control was selected from women attending CPGP of SSGH Hospital for any complaints, health problems other than obstetrics and gynecology.

In order to evaluate the role of abortion as a risk factor, which might influence the pathogenesis of PID, each patient was matched with a patient in the concurrent comparison group with respect to the age (by 5 years’ age group) history.

Numerous risk factors, markers, or both have been associated with PID. Among these are young age, a current STD infection or a previous gonococcal infection, multiple sex partners, and the use of an intrauterine contraceptive device.[2]

So to control confounders, we have kept following exclusion criteria:

1. Those who had multiple sexual partners were excluded from cases as well as controls.
2. Copper-T users and condom users were excluded from cases as well as controls.

Data were entered and analyzed by Epi-Info.

The odds ratio for PID with natural abortions as a risk factor was 3.52 (95% CI: 1.81–6.83%). This suggests an etiological fraction of 71.65% for PID (95% CI: 44.8–85.4%) among natural abortions (*P* = 0.0002) [Table 1]. The odds ratio for induced abortions, as a risk factor, was 2.09 with the 95% CI 0.99–4.40%. An induced abortion was not significantly higher in cases than controls (*P* = 0.073). It may be due to the small sample size.

**Table 1:** Association between natural abortion and pelvic inflammatory disease

	Total abortions among cases	Total abortions among controls	Total
Natural abortions present	38 (26.58%)	14 (9.33%)	52
Natural abortions absent	105 (73.42%)	136 (90.60%)	241
Total	143	150	293

We have considered natural abortions as a risk factor because of retained product of conception and excessive bleeding. Both are favorable media for the growth of bacteria; moreover, in women who are already moderate to severely anemic, further blood loss would enhance chances of infection. In induced abortions, external instrumentation and manipulation also increases chances of infection.

Because of insufficient choice of modern contraception, poor access to quality abortion service, including untrained health providers with inadequate knowledge of modern induced abortion techniques, the practice although legal can still be a serious health problem. Typically, this appears to be the situation in our country.

In the present study, we cannot say that abortions cause PID or PID causes abortions. We can only say that an association exists and it does not mean any causal relationship. One can only say that risk of PID is increased with abortions and this difference is statistically significant.

A study was done by Dalaker regarding early complications of induced abortion in primigravidae and it was noticed that the overall rate of PID after the abortion was 4.1%.[4]

A study was conducted by Gogate among women presenting to clinics in Mumbai (India) for pelvic pain and it was found that 26% with confirmed PID reported to have undergone an abortion as compared to 2% of women without PID.[3]

So natural abortions are associated with PID.
